# RF Channel-Selectivity Sensing by a Small Antenna of Metamaterial Channel Filters for 5G Sub-6-GHz Bands

**DOI:** 10.3390/s20071989

**Published:** 2020-04-02

**Authors:** Muhammad Kamran Khattak, Changhyeong Lee, Heejun Park, Sungtek Kahng

**Affiliations:** Department of Information and Telecommunication Engineering, Incheon National University, Incheon 22012, Korea; k.khattak@huawei.com (M.K.K.); Antman@inu.ac.kr (C.L.); h.park.inu@gmail.com (H.P.)

**Keywords:** 5G mobile communication, Sub-6-GHz, compact antenna, channel selection, channel filters, metamaterials

## Abstract

In this paper, a new small antenna is suggested for 5G Sub-6-GHz band mobile communication. It can change the channel among the three given bands (called the 3.5-GHz area), as a wide-band antenna is connected to a small multiplexer comprising three metamaterial channel filters. The function of channel selection of this antenna system is experimentally demonstrated to prove the validity of the presented scheme. The channel selection for 5G mobile communication is conducted from *f*_1_ (channel 1) through *f*_2_ (channel 2) to *f*_3_ (channel 3), when TX and RX antennas with gains over 0 dBi and *S*_11_ less than −10 dB are located far-field apart (R_Far_ ≫ 2.1 cm), and result in the transmission coefficient (*S*_21_) being the greatest at the selected channel, which is detected by a vector network analyzer.

## 1. Introduction

Over half of the last decade, 5G has been a catchphrase in IT industry with another that is the 4th industrial revolution. It has been driving the world’s first-class IT institutes to translate its hidden potential use-cases into practices in the shape of IoT devices, driverless cars, faster cell-phone-featuring networking services, and so on. It is a matter of course that RF components and antennas corresponding to the given bands are necessary to these wireless devices [1,2]. 

As a way to increase the speed of data transfer, the use of millimeter-wave areas was suggested [3,4] and connectivity had to be added. Hence, beamforming becomes a basic virtue to millimeter-wave antennas which are made as part of a chip for controlling the beam from an array. Besides beam-control, a wide-band is expected from the antenna to take care of multiple wireless network service providers as one device. The 5G spectrum is grouped into millimeter-waves and sub-6-GHz bands, and each group is divided into three channels. As to the sub-6GHz-group or simply speaking, the frequency range, f = 3.5 GHz area which consists of three channels from frequencies f = 3.46 to 3.65 GHz, when the antenna is purchased with a wide-band, it should be able to distinguish one channel from another for being adaptive to the change in the mobile environment.

A channel is distinguished from others with a filter in the wireless communication system. In spite of there being many RF filters of the conventional design methods for wireless systems, intrinsic limitations are given to size-reduction and extension to an upper level circuit combining or dividing channels. One cause of the limitations is the half-wavelength resonance condition. This limitation is eased by the metamaterial filter design method which can minimize the size of the structure and improve the passband and stopband characteristics [5,6]. G. Jang et al. showed a sub-wavelength-sized filter of the zeroth order-resonance (ZOR) effect of the CRLH TX-line, which has a lower loss and a wide-stopband [5]. C. Liao made a filter of a new shape which obtains a stronger coupling in a limited area [6]. On the contrary to the metamaterial configuration for smaller geometries, the size of a filter is reduced by curling a straight stepped transmission-line resonator (SIR) and adding stubs [7,8,9,10,11,12,13,14,15]. Hou et al. folded SIRs and attached stubs to have multiple bands such as 2.13 and 3.47 GHz [7]. Similarly, Sun et al. used SIRs to generate multi-mode resonance (MMR) at harmonic frequencies of 1.5 GHz [8]. Like them, Cho and Chen utilized SIRs flanked by TX-lines for the generation of triple bands [9,10]. Xu, Zhang, and Chu short-ended one of the MMRs to reduce the area of triple-band filters [11,12,13]. MMRs are used only as ports and enclose smaller resonators to make passbands at 2.3, 3.7, and 5.3 GHz [14]. Li et al. coupled two MMRs with gaps to resonate at 1.8, 2.4, and 5.8 GHz [15]. The passbands of each are spaced apart with large frequency gaps. In addition, these kinds of dual- or triple-band filters alone cannot distinguish channels effectively if the channels are closely located side by side. Selectivity in the contiguous channel case can be realized as the following. Multiplexers like a duplexer and more channel elements are made up with filters and make the system effectively choose a channel [16,17,18]. One example of this kind is introduced in M. Chen et al.’s work where the conventional filters are assembled [16]. Tantiviwat et al. showed a multiplexer comprising stub-loaded MMRs as non-metamaterial filters and coupled port, one with two filters as parallel-edge coupling unlike the junction-type multiplexer [17]. Open-gapped SIR ring filters for effective size-reduction gathered to the common TX-line to implement a triplexer [18]. Meanwhile, new metamaterial filters are adopted as channel elements and put together along a line merged to the common port [19].

In this paper, a 3.5-GHz-area wide-band antenna is grafted onto a new metamaterial multiplexer to distinguish received signals to different sub-6-GHz channels. Channel selectivity can be given by a stacked structure like an antenna that has multiple bands like [20,21], but they have radiating elements modified with slits which may cause the tilted far-field patterns at a frequency, and their bands are not closely located and cannot cover the 5G service. Different from them, separately designed filters and multiplexers are combined with the wide-band antenna without slits which will distort the beam. The channels of the multiplexer are secured by three metamaterial bandpass filters consistent with the three mobile service providers’ frequencies. The ability of channel selection is checked by a test setup where all the ports of the filter, multiplexer, and antenna are set at the 50 Ω impedance and a wide-band metamaterial array antenna as the TX from [22] sends the signals of the three channels over the 3.5 GHz area and the aforementioned triplexer-fed wide-band antenna as the RX receives the signals of frequencies *f*_1_, *f*_2_, and *f*_3_ at the common port and pushes them to their own channel filter ports. The TX and RX antennas with gains greater than 0 dBi and S_11_ below −10 dB in the operational bands can make a wireless link for a full-band of the 5G sub-6-GHz communication.

## 2. TX and RX Antennas with Novel Components

**Figure 1:**
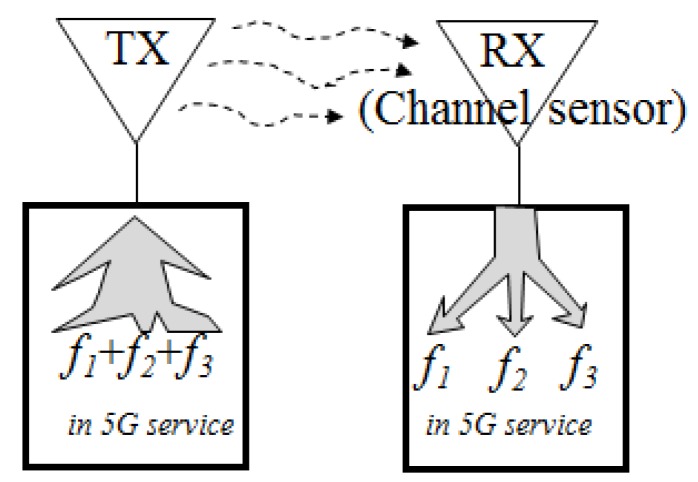
Three channel signals combined in the TX antenna will be split and selected to their corresponding bands in the RX antenna system.

The wireless communication link for the 5G sub-6-GHz band is investigated between the TX and RX antennas in the proposed manner depicted as below in Figure 1.

The signals from the three bands of the 3.5 GHz region for the 5G service, say, *f*_1_, *f*_2_, and *f*_3_ are transmitted from the TX side to the RX side. The received frequencies *f*_1_, *f*_2_, and *f*_3_ are split into channels 1, 2, and 3 each, which is channel selection.

**Figure 2:**
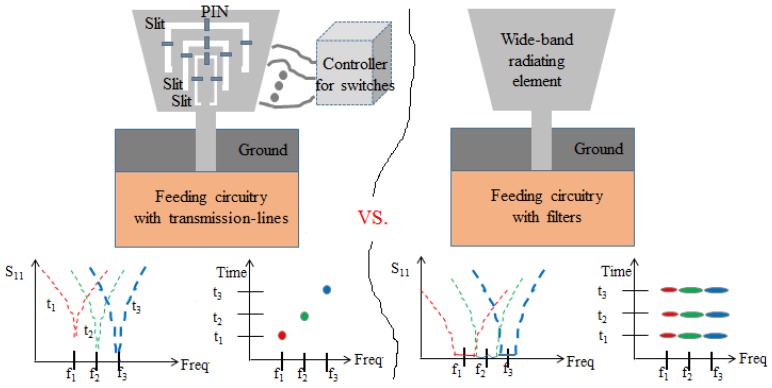
Two schemes of channel selection: switch-loaded RX antenna with the ordinary feed (**Left**), and non-active device loaded RX antenna with the multiplexer feed circuit (**Right**).

The left schematic of Figure 2 is the antenna system loaded with switches, where S_11_ is the reflection coefficient in the figure. The wide-band radiating element has multiple slits where PIN diodes are attached. The PIN diodes are turned on by the controller and DC bias circuit to catch one signal among frequencies *f*_1_, *f*_2_, and *f*_3_. Synchronization with the TX is required and probabilistic. The center frequency, not the bandwidth, of each band is decided, and one frequency is usable at a time. To overcome such problems in realization and operational cost, the right schematic is suggested. The radiating element is not loaded with active components. It should be a wide-band and compact structure. The feed circuitry should have a multiplexer that divides the collection frequencies into different bands through filters. Then, the bandwidth can be controlled with the center frequency. Synchronization is not needed, which enables all three bands to be used, regardless of moments in time. Also, small filters are required to make the multiplexer small. The channel filter is realized as a metamaterial structure as follows.

**Figure 3:**
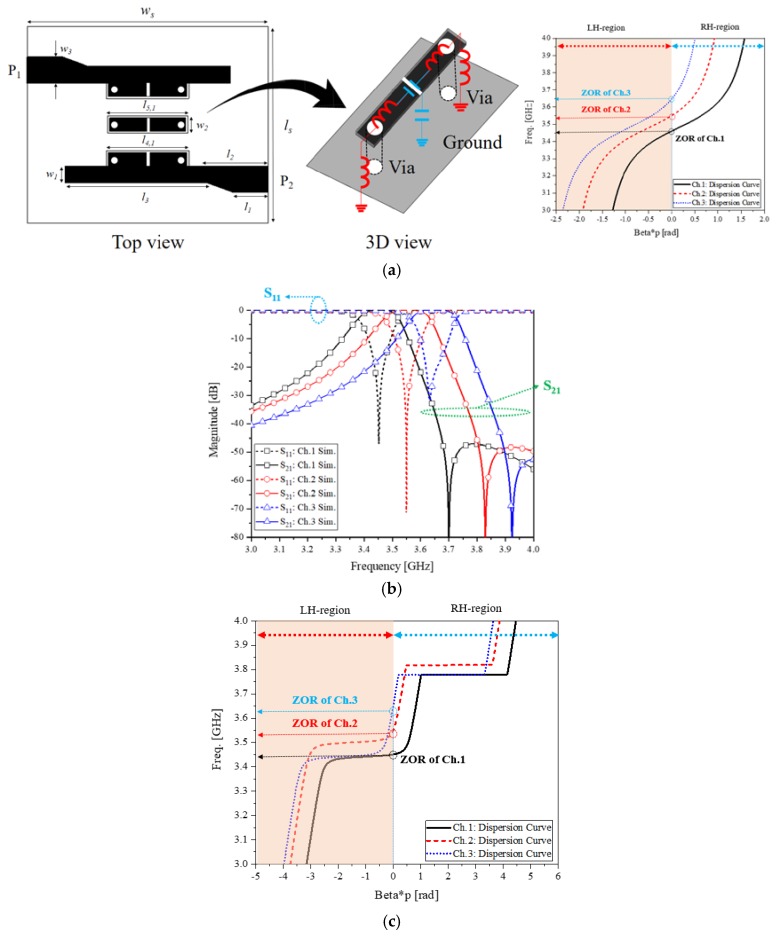
The metamaterial channel filter (**a**) Geometry and equivalent circuit (**b**) Frequency responses of the three bands (**c**) Dispersion diagram of the channel filters as the metamaterial property.

In Figure 3, the shape of the proposed channel filter and its performances are given. First of all, the channel filter has a gate-shaped resonator comprising a short strip on FR4 substrate and vias. The strip as the series inductance, its gap as the series capacitance, the via as the shunt inductance along with the shunt capacitance between the ground and strip can make a CRLH metamaterial resonator. The equivalent-circuit of this CRLH resonator is handled to create the ZOR at the center frequencies of the channels, as in Figure 3a. At this moment that a relatively large series capacitance is needed, the gap looks very small compared to the other parts of the 3D structure. Second of all, the resonators are coupled from the input port through the middle part to the output port to work as a filter. Other metamaterial filters use horizontal SRRs or align longitudinal resonators, but the new filter uses vertical structures and cascade them transversely between the ports. The physical dimensions of the filter are *l_s_*, *l*_1_, *l*_2_, *l*_3_, *l*_4,1_, *l*_4,2_, *l*_4,3_, *l*_5,1_, *l*_5,2_, *l*_5,3_, *w_s_*, *w*_1_, *w*_2_, and *w*_3_ set as 19.3, 3.5, 6, 14, 7.75, 7.4, 6.9, 7.5, 7.2, 6.7, 23.7, 1.7, 1.3, and 2.6 in mm for Figure 3a. These values are put in Table 1 and used for one channel and tuned to form the three channels. Figure 3b shows the passbands centered at 3.46, 3.55, and 3.65 GHz, compliant with the industrial requirement. Each channel’s S_11_ is less than −10 dB and S_21_ is close to −0.5 dB in operating frequency bands. The ZOR occurs coinciding with frequencies *f*_1_, *f*_2_, and *f*_3_, along with the LH- and RH-regions in the dispersion diagram presenting the metamaterial characteristics of the geometry. These filters are assembled by the following multiplexer.

**Figure 4:**
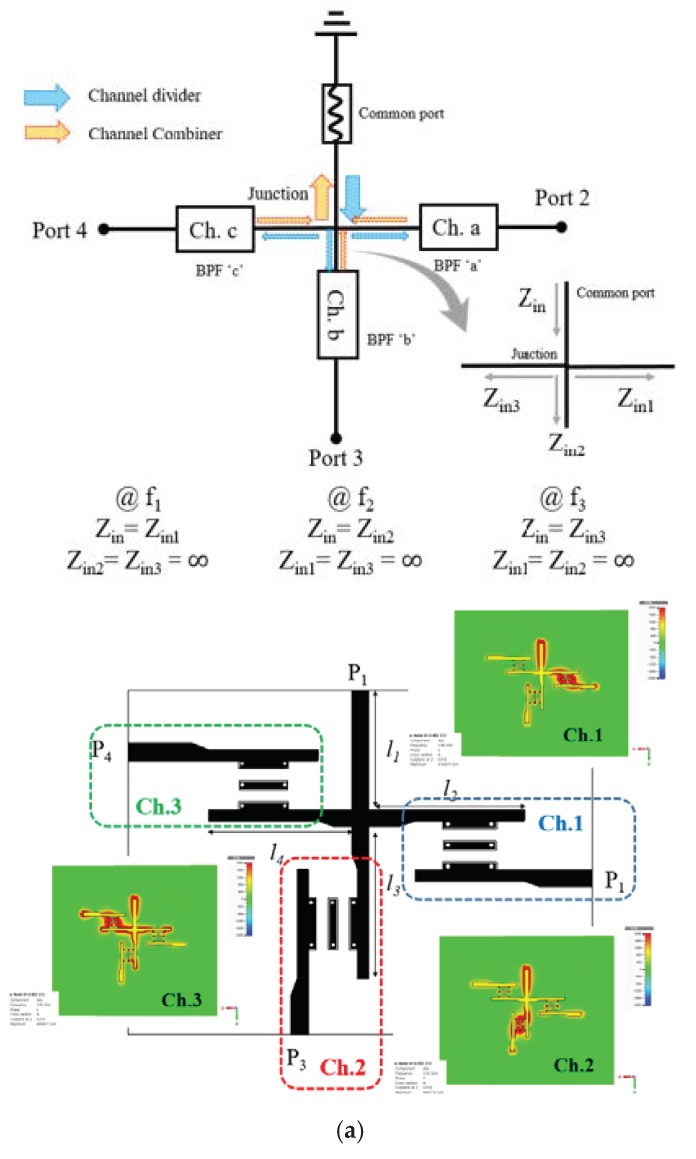

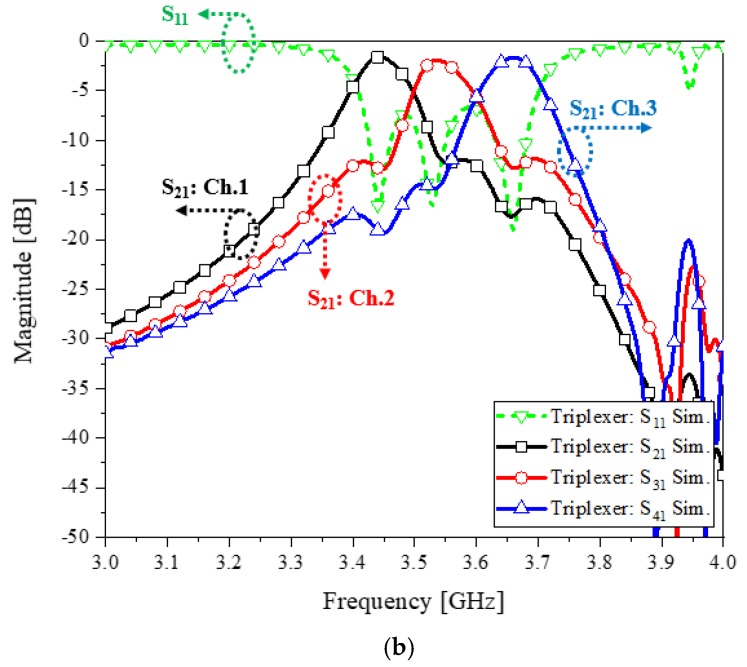
The compact multiplexer comprising the three channel filters. (**a**) Geometry. (**b**) The overall frequency response of the three bands.

As mentioned with Figure 2, a multiplexer is needed to combine the channels. Figure 4a shows the schematic and geometry of the multiplexer where P_1_ is the port connected to the RX antenna to receive all the signals having an electrically small footprint. The schematic describes the signal flow from P_1_ to the impedance-matched branches. Its geometrical parameters *l*_1_, *l*_2_, *l*_3_ and *l*_4_ are 18, 23.5, 22.9, and 21.5 in mm with FR4 substrate, respectively. In order to keep the area from being larger, a multi-branch junction shape is chosen and the positions of the channel branches are determined to retrieve all the passbands the same as individual channel filters. The P_2_, P_3_, and P_4_ are output ports of *f*_1_, *f*_2_, and *f*_3_, in that order. Figure 4b presents the passbands obtained at the ports P_2_ for Ch. 1, P_3_ for Ch. 2, and P_4_ for Ch. 3. Also, the electric field distributions as the insets prove that only the right frequency signal enters the channel. The results are consistent with those in Figure 3b. It is time to mention wide-band antennas for transmitting and receiving covering the three bands.

**Figure 5:**
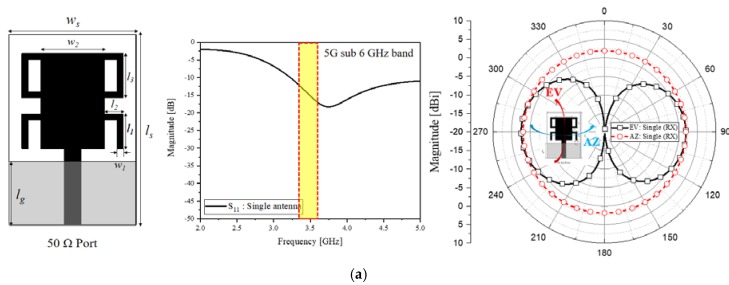

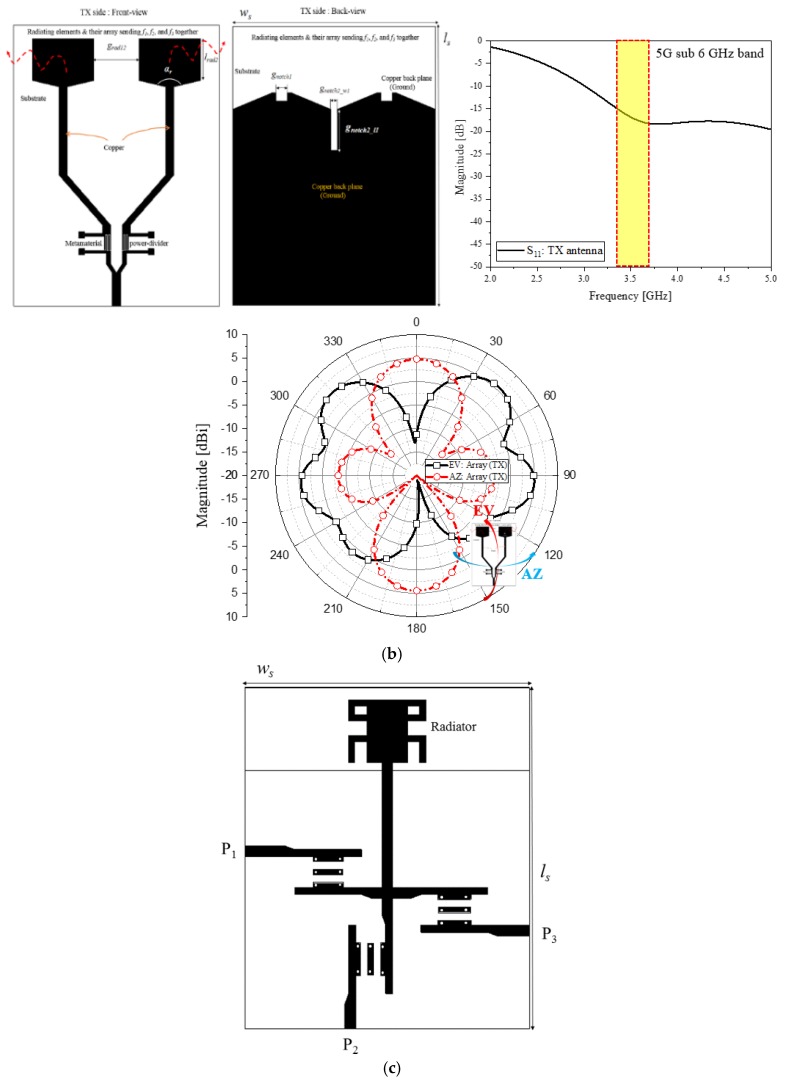

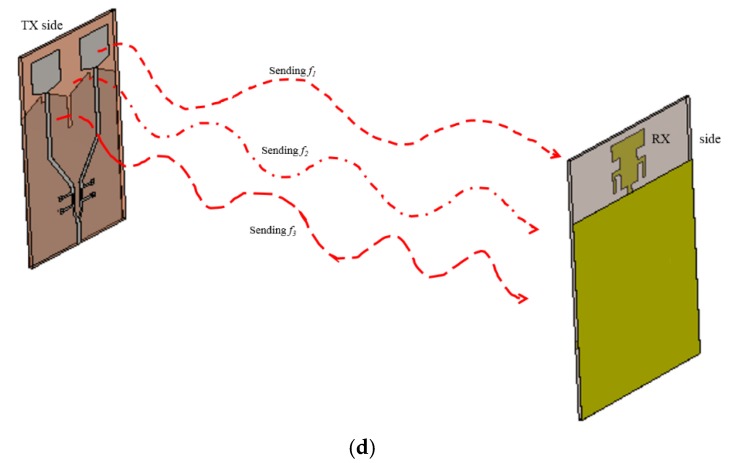
Shapes and interactive configuration of the TX and RX antennas. (**a**) A compact wide-band RX antenna and far-field patter for signal. (**b**) Front and bottom views of the wide-band TX antenna. (**c**) Wide-band RX antenna connected to multiplexer of three channel filters. (**d**) Scheme of linking the TX and RX antennas.

Figure 5a,b are a wide-band RX antenna and an ultra-wide-band array TX antenna. The TX antenna is adopted by the authors’ former work to have an increased directivity [22]. The TX antenna elements which consist of a pentagonal radiator and a triangular ground with a dent for impedance match and show a wide-band impedance match, as shown in S_11_ of Figure 5b, stem from a metamaterial power-divider. This arraying is intended to increase the directivity of the azimuth-plane radiation-pattern as appearing in Figure 5b. Meanwhile, the RX antenna should have an omni-directional radiation to respond to an arbitrary angle of incidence and have a wide-band function from 3.2 to 5.5 GHz as S_11_ of Figure 5a. Explaining the geometry, Figure 5a as a stub-loaded stepped patch for RX has 30, 10, 5.5, 3, 7, 20, 1 and 10 mm as *l_s_*, *l_g_*, *l*_1_, *l*_2_, *l*_3_, *w_s_*, *w*_1_, and *w*_2_ with FR4 substrate, respectively, and is fed by a multiplexer of three metamaterial channel filters. *g_rad12_*, *l_rad2_*, *g_notch1_*, *g_notch2_w1_*, *g_notch2_l1_*, *w_s_*, *l_s_*, *l_cmch1_*, *l_cmch2_*, *l_cmch3_*, *l_ch1-reso1_*, *l_ch1-reso2_*, *g_ch1-12_*, *g_ch1-23_*, *w_ch1-reso1_*, *w_ch1-reso2_*, *w_s_*, and *l_s_* are 11.7, 10, 3, 1.75, 10, 53.7, 67.5, 34.2, 56, 29.6, 7.1, 6.9, 2, 2, 1, 1, 70, and 82 in mm, respectively, with Figure 5c. The values are shown in Table 1. As another parameter, *α_r_*, which is the angle of two tapering sides of the TX antenna element, is set at 160.6°, which needed to match the impedance over the wide-band. Figure 5d shows how the two blocks are placed a far-field distance apart.

## 3. Experiment of Channel Selectivity Sensing

The components and circuits above are manufactured. From the filters of the multiplexer to antennas, power transfer coefficients are investigated.

**Figure 6:**
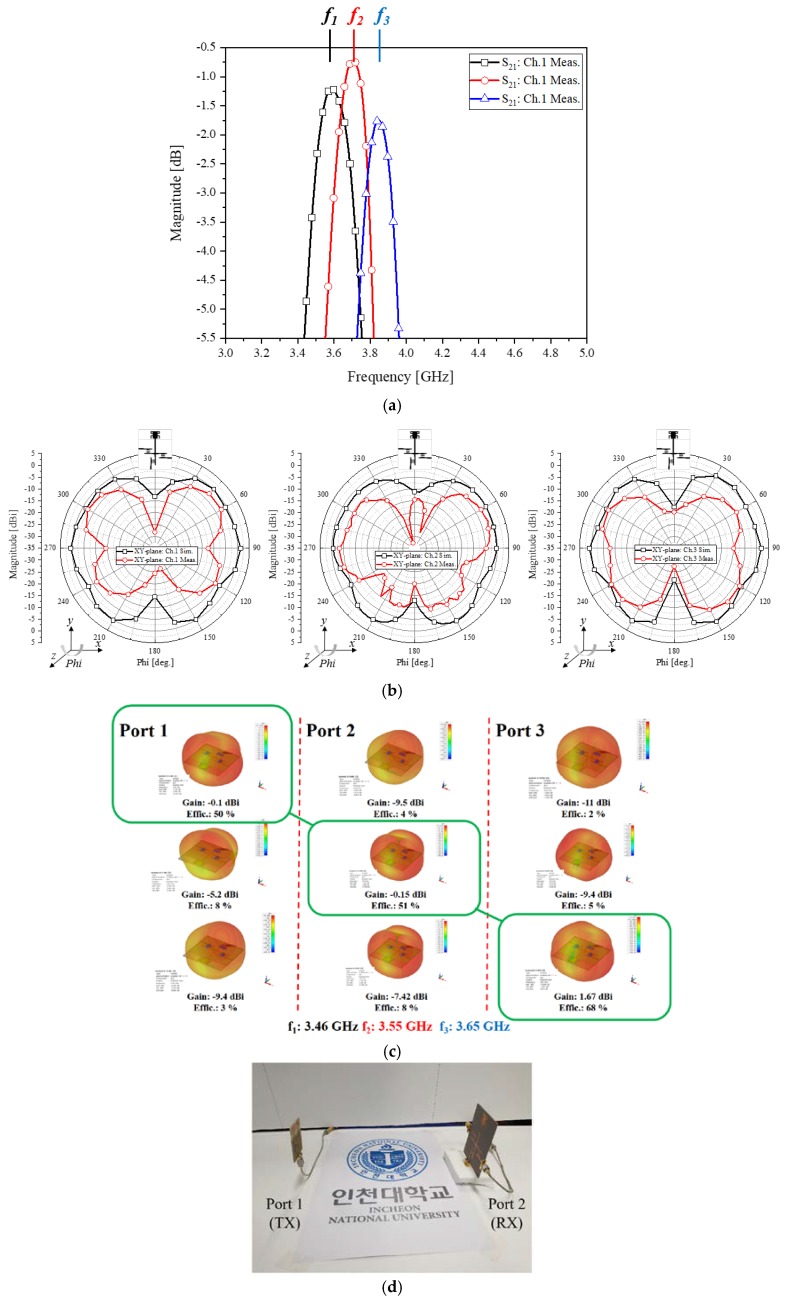

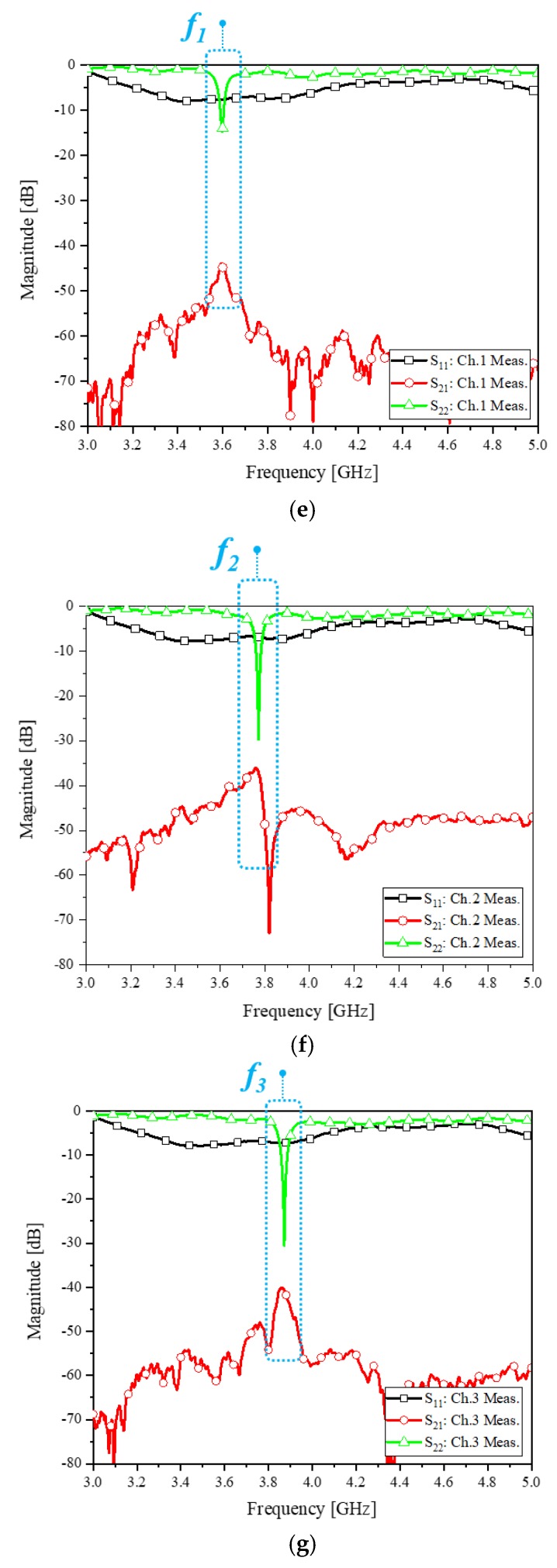
Test results of channel selection (**a**) Measured passbands of the filters (**b**) Simulated and measured far-field patterns of the RX antenna at *f*_1_, *f*_2_, and *f*_3_ (**c**) Antenna gains of the RX antenna with columns as TX signals and rows as RX channels (**d**) Photograph of the realistic the TX to RX antenna wireless link test-setup (**e**) Antennas’ S_ii_ and received *f*_1_ signal (**f**) Antennas’ S_ii_ and received *f*_2_ signal (**g**) Antennas’ S_ii_ and received *f*_3_ signal.

Throughout the test, there are no amplifiers for both the TX and RX blocks. Before sensing each channel of the wireless link, the measured functions of the filtering blocks of the multiplexer are looked into, Figure 6 shows the three passbands of the 5G sub-6-GHz area. Frequencies *f*_1_, *f*_2_, and *f*_3_ are shifted from 3.46, 3.55, 3.65 GHz on account of manufacturing errors and connector assembly errors. However, it is revealed that the errors pose insignificant problems in observing the channel selectivity of the system. The measured far-field pattern of the RX antenna fed by the multiplexer, when each of the three channels is turned on, is plotted in comparison with its simulated version as in Figure 6b. There is a matrix of the beam-patterns of the RX channels with respect to the TX channels in Figure 6c. Each column of the matrix means a TX signal and each row implies an RX channel. For example, as for the first column as TX *f*_1_, the first row as RX channel 1 has the greatest antenna gain and efficiency over the entire elements of the first column. Hence, the diagonal elements outperform the rest of the matrix. The co-channel link works much better than cross-channel links. For the final experiment, the filters and multiplexer are adopted to the RX block which realistically faces the TX block as seen in Figure 6d. Figure 6e–g present a channel chosen in turn from channel 1 to channel 3, the impedance of the RX antenna is matched to the chosen frequency (S_22_) and only accepts the desired frequency signal out of many incident frequencies emanated from the ultra-wide-band (S_11_) of the TX. The peak of the red curve as S_21_ (power transfer coefficient) appears at frequencies *f*_1_, *f*_2_, and *f*_3_ by taking turns, as a proof of the RF link and proposed channel-selection scheme. Besides, the contributions of this work are mentioned in Table 2 where the differences of this work and the referenced works are compared.

## 4. Conclusions

For the 5G sub-6-GHz communication, a small antenna system is introduced to have the ability of channel selection to avoid the interference from other neighboring channels. The channel is distinguished by devising a compact multiplexer of three metamaterial channel filters. A TX antenna as an ultra-wide-band metamaterial array and the proposed antenna as the RX are put along to check the wireless links between them and the channel-selection function. According to the experiment, change from frequencies *f*_1_ through *f*_2_ to *f*_3_ is detected clearly by the RX side. This will lead to a way to check the channel selection ability of 5G mobile services.

## Figures and Tables

**Table 1:** Geometrical parameters of the filters, multiplexers and antennas.

**Filters**									
Items	*l_s_*	*l*_1_	*l*_2_	*l*_3_	*l*_4,1_	*l*_4,2_	*l*_4,3_		
Val. (mm)	19.3	3.5	6	14	7.75	7.4	6.9		
Items	*l*_5,1_	*l*_5,2_	*l*_5,3_	*w_s_*	*w*_1_	*w*_2_	*w*_3_		
Val. (mm)	7.5	7.2	6.7	23.7	1.7	1.3	2.6		
**Multiplexers and Antennas**									
Items	*l_s_*	*l_g_*	*l*_1_	*l*_2_	*l*_3_	*w_s_*	*w*_1_	*w*_2_	
Val. (mm)	30	10	5.5	3	7	20	1	10	
Items	*g_rad12_*	*l_rad2_*	*g_notch1_*	*g_notch2_w1_*	*g_notch2_l1_*	*w_s_*	*l_s_*	*l_cmch1_*	*l_cmch2_*
Val. (mm)	11.7	10	3	1.75	10	53.7	67.5	34.2	56
Items	*l_cmch3_*	*l_ch1_reso1_*	*l_ch1_reso2_*	*g_ch1-12_*	*g_ch1-23_*	*w_ch1-reso1_*	*w_ch1-reso2_*	*w_s_*	*l_s_*
Val. (mm)	29.6	7.1	6.9	2	2	1	1	70	82

**Table 2:** Comparing the differences of the proposed work and others’.

Ref. No.	Single-Band Selectivity	Contiguous/Ch. Selection	Metamaterial Concept	5G Service Coverage	Multiplexing	Size (λ_g_ @ Center Freq.)
[7]	×	×	×	×	×	0.225λ_g_ × 0.226λ_g_
[8]	×	×	×	×	×	0.16λ_g_ × 0.16λ_g_
[9]	×	×	×	×	×	0.26λ_g_ × 0.36λ_g_
[10]	×	×	×	×	×	0.23λ_g_ × 0.12λ_g_
[11]	×	×	×	×	×	0.18λ_g_ × 0.27λ_g_
[12]	×	×	×	×	×	0.108λ_g_ × 0.521λ_g_
[13]	×	×	×	×	×	0.08λ_g_ × 0.15λ_g_
[14]	×	×	×	×	×	0.44λ_g_ × 0.39λ_g_
[15]	×	×	×	×	×	0.16λ_g_ × 0.17λ_g_
[17]	×	×	×	×	×	0.18λ_g_ × 0.20λ_g_
[21]	×	×	×	×	×	0.75λ_0_ × 0.75λ_0_
This work	○	○	○	○	○	* 0.32λ_g_ × 0.41λ_g_ ** 1.19λ_g_ × 0.89λ_g_

* Filter: Ch.1; ** Triplexer; ○ means Yes; X means No.
